# Comparative pathophysiology and molecular insights into cutaneous and non-cutaneous canine skin cancers: focus on melanoma, mast cell tumors, and squamous cell carcinoma

**DOI:** 10.3389/fimmu.2025.1624598

**Published:** 2025-11-27

**Authors:** Sorin Marian Mârza, Camelia Munteanu, Ionel Papuc, Robert Cristian Purdoiu

**Affiliations:** 1 Faculty of Veterinary Medicine, University of Agricultural Science and Veterinary Medicine, Cluj-Napoca, Romania; 2 Biology Section, Faculty of Agriculture, University of Agricultural Sciences and Veterinary Medicine, Cluj-Napoca, Romania

**Keywords:** canine skin cancer, mast cell tumors, melanoma, squamous cell carcinoma, veterinary oncology, tumor biomarkers, immunotherapy, tyrosine kinase inhibitors

## Abstract

Skin cancer is one of the most frequently diagnosed neoplasms in dogs, encompassing a range of malignancies with significant clinical implications. Among them, mast cell tumors (MCTs), melanomas, and squamous cell carcinomas (SCCs) signify the most common and clinically relevant types, each posing distinct therapeutic challenges and exhibiting pathophysiological mechanisms. MCTs, accounting for approximately 21% of canine skin tumors, are often driven by mutations in the KIT proto-oncogene, leading to an uncontrolled proliferation of mast cells. Melanomas, while typically benign in cutaneous forms, exhibit aggressive behavior in oral and digital locations, with BRAF and NRAS mutations playing an integral role in tumor growth. Furthermore, SCCs, primarily associated with chronic ultraviolet (UV) radiation exposure, demonstrate significant genomic modifications, including mutations in TP53 and increased expression of COX-2, resulting in carcinogenesis. Accurate diagnosis of these tumors significantly relies on cytology, histopathology, and immunohistochemical markers. Moreover, advanced imaging techniques such as computed tomography (CT) and positron emission tomography (PET) can potentially enhance staging and prognostication. Treatment modalities vary based on tumor type and stage, including surgical excision, radiation therapy, chemotherapy, and emerging targeted therapies. Tyrosine kinase inhibitors (TKIs), such as toceranib phosphate (Palladia) and masitinib, have demonstrated efficacy in MCTs. Likewise, immunotherapies, including the *Oncept* melanoma vaccine and checkpoint inhibitors, offer novel therapeutic avenues. Comparative oncology continues to underscore molecular similarities between canine and human skin cancers, advancing translational research and developing precision medicine techniques in veterinary oncology. This review comprehensively synthesizes state-of-the-art literature on canine skin cancer, addressing pathophysiological mechanisms, diagnostic advancements, and emerging therapeutic strategies. In addition, this review aims to improve early detection, treatment outcomes, and enduring prognosis for affected canines by integrating recent findings into molecular oncology and comparative medicine.

## 1 Introduction

Skin cancer is one of the most frequently diagnosed neoplasms in dogs, with significant implications for veterinary oncology. Among the broad range of diverse skin tumors observed in canines, mast cell tumors (MCTs), melanoma, and squamous cell carcinoma (SCCs) specify the most commonly witnessed and clinically significant types. According to an estimate, skin tumors account for approximately 20–30% of all canine neoplasms, signifying their prevalence in veterinary practice (1–3). Among them, MCTs are predominantly noticeable, constituting up to 21% of all canine skin tumors. These tumors show diverse biological behaviors, ranging from benign forms to aggressive malignancies. Their diagnosis typically relies on cytological evaluation and histopathological grading, with prognostic factors including mitotic index and cellular markers (4).

Furthermore, melanoma specifies a significant subset of canine skin cancer. While these cancer types are often discovered in mucocutaneous regions, they also manifest themselves on the skin, where their behavior varies from benign to highly metastatic forms. Advances in molecular oncology have determined multiple similarities between canine and human melanoma (5, 6), accentuating the translational potential of canine models for evaluating tumor biology and therapeutic development (7, 8). Other types of tumors (MCTs, SCCs) can be explained further here to balance the major emphasis on melanomas. Another significant type of skin cancer, cutaneous squamous cell carcinoma, originates primarily due to ecological factors, such as chronic exposure to ultraviolet (UV) radiation. These tumors are typically observed in lightly pigmented dogs and those with sparse hair coats. Molecular studies have demonstrated the role of E-cadherin and syndecan-1 as potential markers for tumor growth in canine squamous cell carcinoma, offering in-depth insights into its pathogenesis (9). Early detection of skin cancer in dogs is vital for improving therapeutic outcomes. Moreover, diagnostic tools, *e.g.*, cytology, histopathology, and advanced imaging techniques like elastography, have facilitated earlier and more accurate detection of malignant tumors (10). Additionally, morphologic grading is a cornerstone of tumor assessment, particularly in MCTs, where survival time is closely associated with tumor grade (11). A systematic review of mitotic activity in canine tumors further underscores its importance as a prognostic marker across various neoplasms (12). Melanomas are particularly noteworthy clinically because of their high metastatic potential, aggressive biological behavior, and utility as comparison models in human oncology. Canine skin malignancies account for a significant proportion of neoplastic diseases in veterinary medicine. Developing more potent treatment strategies requires understanding the intricate relationships between tumor cells and the immune system. Specifically, developments in immunotherapy and mechanisms of tumor immune evasion have become major topics in contemporary research, offering translational insights applicable to both human and veterinary medicine. This review offers a comprehensive overview of dogs’ most common types of skin cancer, focusing on their epidemiology, diagnostic approaches, treatment options, and molecular insights. In addition, this study contributes to the evolving strategies for managing canine skin cancer in veterinary practice by exploring recent advancements and limitations in current literature.

## 2 Materials and methods

This review was conducted to synthesize the existing literature on skin cancer in dogs, focusing on common tumor types, diagnostic techniques, treatment options, and prognostic factors. A systematic approach was utilized to specify and evaluate relevant studies from reputable sources. For instance, the literature search was conducted using prominent databases, such as PubMed, Scopus, Web of Science, and Google Scholar. Relevant studies were retrieved by using specific search terms, including combinations of the following keywords: “canine skin cancer,” “mast cell tumors (MCTs) in dogs,” “canine melanoma,” “cutaneous squamous cell carcinoma in dogs,” “diagnostic methods for canine tumors,” and “treatment of canine skin tumors.” Furthermore, articles published in English between 1990 and 2024 were included to capture both foundational and recent studies in this research area.

The inclusion criteria for the review emphasized whether the studies addressed the diagnosis, treatment, or pathophysiology of canine skin cancer. Studies focusing on MCTs, melanoma, or SCCs in dogs were prioritized. Moreover, only peer-reviewed articles published in reputable journals were incorporated. On the other hand, the exclusion criteria focused on whether the studies were not specific to dogs, such as those evaluating different species without canine relevance. Non-peer-reviewed studies, such as conference abstracts and opinion pieces, were also excluded. Likewise, any research studies lacking sufficient methodological detail or those with limited sample sizes were eliminated.

Data were extracted from each selected study to specify key findings associated with the pathophysiology, diagnostic techniques, and therapeutic approaches for canine skin cancer. Moreover, information on novel insights into molecular mechanisms and emerging treatment methods was also collected. Next, the selected studies were categorized into different themes based on tumor types, including MCTs, melanoma, and SCCs. Additional sections were created to explore diagnostic advancements, therapeutic options, and emerging research in molecular and comparative oncologies.

This review survey has certain limitations. For instance, only English-language studies were included, which may have overlooked many relevant studies published in other languages. Additionally, the reliance on electronic databases may have resulted in the omission of older studies that are available only in print. However, despite these limitations, this review thoroughly summarizes the current understanding of canine skin cancer. The images were created in BioRender. Munteanu, C (2025). https://BioRender.com/em1czad.

## 3 Pathophysiology of canine skin cancer

Skin cancers in dogs, particularly MCTs, melanomas, and SCCs, exhibit distinct pathophysiological mechanisms influencing their behavior and clinical outcomes. This section explores these tumors’ cellular and molecular foundations, supported by evidence from recent scientific studies.

### 3.1 Mast cell tumors

MCTs are dogs’ most frequently diagnosed malignant skin tumors, accounting for up to 21% of all cutaneous neoplasms (13, 14). These tumors originate from dermal mast cells, which are critical in dogs’ allergic reactions and immune responses (15). The pathogenesis of MCTs is often associated with specifically, internal tandem duplications (ITDs) in exon 11 of the KIT gene that lead to constitutive activation of the receptor, promoting unchecked mast cell proliferation and survival (16).

MCTs in dogs exhibit an extensive range of biological behaviors, from indolent to highly aggressive forms. Therefore, histopathological grading systems, such as the Patnaik and Kiupel systems, are broadly employed to predict clinical outcomes. High-grade tumors exhibit attributes like high mitotic (17) indices, multinucleation, and nuclear pleomorphism, all of which are associated with poor prognosis. Moreover, neoplastic mast cells release bioactive substances like histamine, heparin, and proteases, which contribute to local inflammation, edema, and, in severe cases, gastrointestinal ulceration (18). The tumor microenvironment also plays a vital role in MCT growth. Studies have specified increased angiogenesis and vascular endothelial growth factor (VEGF) expression in aggressive tumors, underscoring potential therapeutic targets. A survey by Rassele et al. (2025) assessed VEGF expression in primary canine MCTs and their associated lymph node metastases, finding VEGF expression in 50% of the cases. Although VEGF immunolabeling did not directly correlate with survival times, the VEGF presence exhibits its role in tumor progression and underscores its potential as a therapeutic target (19).

### 3.2 Melanomas

**Figure 1:**
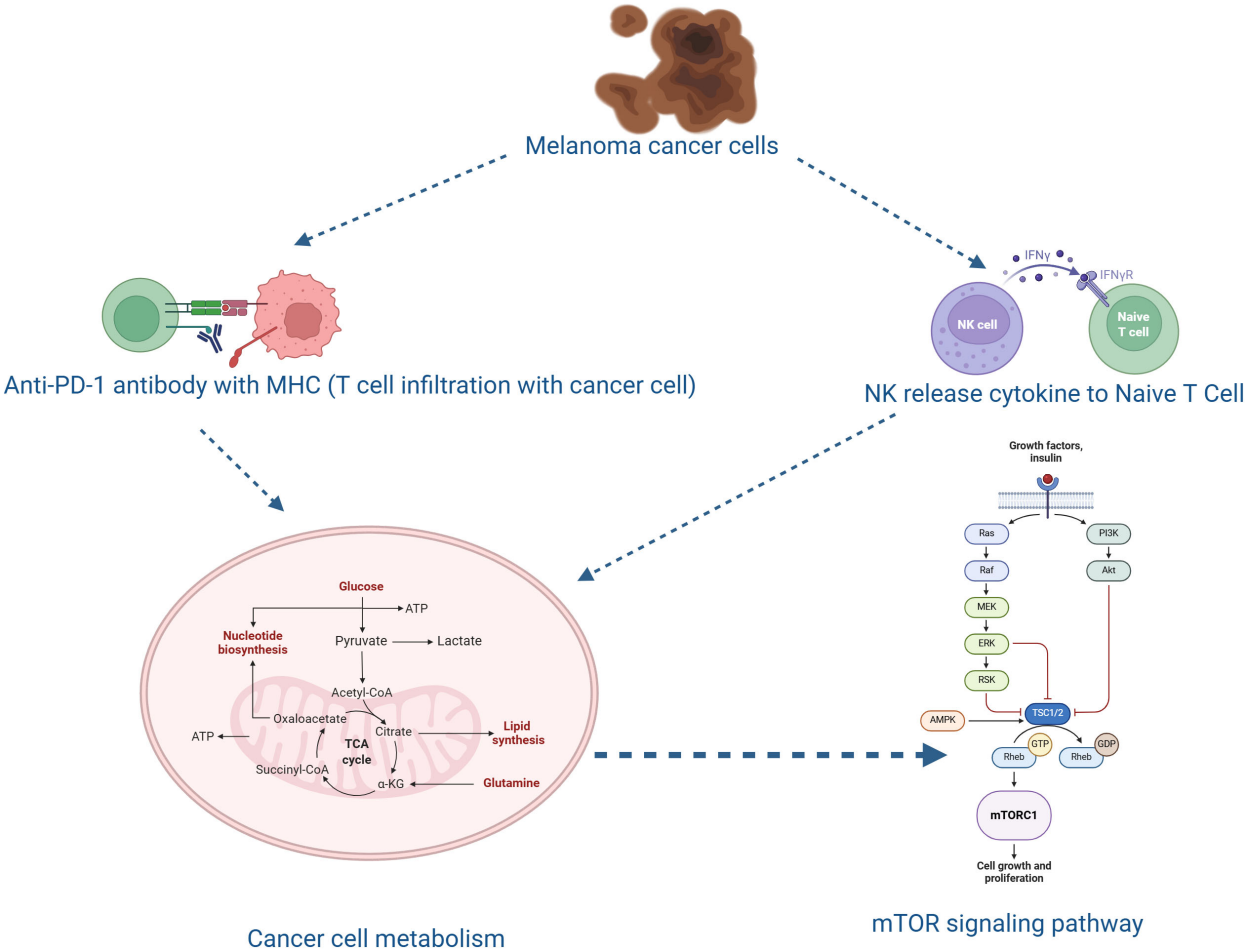
Diagrammatic depiction of the immunometabolic interactions that contribute to the development of canine melanoma and the response to treatment. Melanoma canine cancer cells use metabolic changes and cytokine signaling to regulate immune cell activity. Naïve T cells are activated by interferon-gamma (IFNγ), which is released by natural killer (NK) cells. When an anti-PD-1 antibody targeting the PD-1/MHC axis is present, activated T cells infiltrate the tumor microenvironment and enhance anticancer immunity. Activated T cells, in the presence of an anti-PD-1 antibody targeting the PD-1/MHC axis, infiltrate the tumor microenvironment and promote antitumor immunity. Rapid growth and immune evasion are supported by metabolic processes in cancer cells, including glycolysis, the TCA cycle, and lipid and nucleotide biosynthesis. The mTOR signaling system, which integrates signals from growth factors and nutrients to regulate cell growth and proliferation, is closely linked to these metabolic activities. Together, these mechanisms illustrate the interplay between immune activation, cancer cell metabolism, and signaling pathways in melanoma pathophysiology and therapeutic intervention.

Canine melanomas originate from melanocytes and occur in multiple anatomical locations, such as the skin, oral cavity, and digits (20). However, it is essential to distinguish between the benign cutaneous melanocytomas that represent the majority of canine cutaneous melanocytic tumors and the malignant variants, which include subungual and oral melanomas.While cutaneous melanomas are typically benign, those in the oral cavity and digits are observed to be aggressive and metastatic (20, 21). In fact, there remains some ambiguity in the veterinary oncology literature as to whether digit-related melanomas should be categorized under cutaneous melanomas, a distinction that warrants clarification when discussing canine cutaneous forms. Genetic mutations, such as those in the *BRAF* and *NRAS* genes, are implicated in melanoma pathogenesis. Consequently, they lead to aberrant activation of the MAPK signaling pathway and uncontrolled cellular proliferation (22) (**Figure 1**). The melanomas’ ability to evade immune surveillance is a hallmark of their malignancy. These tumors often express immune checkpoint molecules like programmed death-ligand 1 (PD-L1), which inhibit T-cell-mediated immune responses. In canine oral melanomas, PD-L1 expression has been detected in tumor cells, and its association with programmed death-1 (PD-1) on tumor-infiltrating lymphocytes contributes to immune evasion. This observation, although well-documented in human melanomas, has been increasingly reported in canine oral and subungual malignant melanomas, indicating similar immune escape mechanisms (23, 24). This mechanism is similar to that observed in human melanomas, where PD-L1 expression accelerates tumor progression by suppressing the host’s immune response. Targeting the PD-1/PD-L1 axis with immune checkpoint inhibitors has exhibited good potential in restoring antitumor immunity and specifies a potential therapeutic strategy for treating melanomas in humans and dogs (25, 26). It should be noted, however, that most of the detailed molecular understanding of these pathways derives from human melanoma studies, with only limited but growing veterinary evidence supporting analogous mechanisms in canine malignant melanoma (23, 24). Oxidative stress plays a vital role in transforming melanocytes into malignant melanoma cells. Specifically, elevated levels of reactive oxygen species (ROS) can induce oxidative stress, leading to DNA degradation and mutations that transform melanocytes into malignant melanoma cells. Moreover, the altered redox balance influences vital signaling pathways, fostering cell proliferation, survival, and metastatic potential (27). These EMT-related mechanisms are extensively described in human malignant melanoma and are believed to play comparable roles in canine oral and subungual melanomas, although further veterinary-specific research is required to validate these parallels.

Metastatic progression in melanoma is strongly influenced by epithelial-to-mesenchymal transition (EMT), a biological process that reduces cell adhesion and enhances tumor cell motility. During EMT, epithelial markers such as E-cadherin weaken intercellular connections, while mesenchymal markers like vimentin are upregulated, promoting cytoskeletal reorganization and invasiveness. These molecular changes enable melanoma cells to detach from the primary tumor and invade distant tissues, significantly contributing to metastasis (28). Matrix metalloproteinases (MMPs), particularly MMP-2 and MMP-9, play a crucial role in this process by degrading extracellular matrix (ECM) components, disrupting tissue integrity, and releasing bioactive fragments that promote EMT. Elevated expression of these enzymes has been associated with increased tumor aggression and poor prognosis in melanoma patients. Specifically, MMP-9’s enzymatic breakdown of ECM barriers modulates the tumor microenvironment, facilitating invasion and dissemination. Consequently, targeting MMP-2 and MMP-9 may offer therapeutic potential for inhibiting EMT and metastasis in melanoma The MMP-9’s enzymatic breakdown of ECM barriers modulates the tumor microenvironment to facilitate metastasis (29). In canine malignant melanoma, increased MMP-2 and MMP-9 expression has similarly been associated with enhanced invasiveness, suggesting conserved mechanisms between species (23).

Melanoma cells employ diverse mechanisms to evade immune surveillance, including the overexpression of PD-L1 that binds to PD-1 receptors on T cells, transmitting inhibitory signals, leading to T-cell exhaustion and impaired immune responses. Overexpression of PD-L1, which binds to T-cell PD-1 receptors and transmits inhibitory signals that cause T-cell fatigue and decreased cytotoxic function, is one of the primary mechanisms. Tumor development is facilitated by this interaction, which effectively limits the body’s ability to mount an effective antitumor response. Therapeutically, immune checkpoint inhibitors targeting the PD-1/PD-L1 axis have shown promise in enhancing immune-mediated tumor clearance and restoring T-cell function by blocking this interaction (30). Interferon-gamma (IFN-γ) has two functions in melanoma immunity beyond its role in immune checkpoint regulation. IFN-γ, primarily produced by activated T cells and natural killer (NK) cells, enhances macrophage cytotoxicity and improves antigen presentation by upregulating MHC class I and II molecules. On the other hand, dysregulated or chronic IFN-γ signaling can paradoxically cause adaptive resistance by promoting an immunosuppressive tumor microenvironment and raising PD-L1 expression on melanoma cells. Therefore, under prolonged exposure, the IFN-γ pathway may be a cause of immunological escape as well as a crucial effector mechanism in immune surveillance. Furthermore, melanoma cells utilize multiple strategies to evade immune surveillance, including the secretion of immunosuppressive cytokines like transforming growth factor-beta (TGF-β) and interleukin-10 (IL-10). Moreover, these cytokines suppress the activation and proliferation of cytotoxic T lymphocytes and natural killer (NK) cells, thereby reducing the body’s antitumor immune response (**Figure 1**).

TGF-β and IL-10 contribute to the immunosuppressive tumor microenvironment by inhibiting effector lymphocyte functions and promoting regulatory T-cell activity, further suppressing immune responses against the tumor (31). Besides secreting immunosuppressive cytokines, melanoma cells contribute to immune evasion by introducing regulatory T cells (Tregs) and myeloid-derived suppressor cells (MDSCs) into the tumor microenvironment. Among these cells, Tregs suppress the activation and proliferation of effector T cells, while MDSCs inhibit T cell function and stimulate tumor progression. This accumulation of immunosuppressive cells within the tumor microenvironment significantly reduces effective antitumor immune responses, enabling melanoma growth and metastasis (32). Furthermore, melanoma-associated fibroblasts (MAFs) substantially contribute to immune evasion within the tumor microenvironment by releasing extracellular vesicles (EVs), which carry various bioactive molecules, such as proteins, nucleic acids, and metabolites. These molecules can modulate the function of immune cells. For instance, EVs derived from cancer-associated fibroblasts (CAFs) have been demonstrated to impact tumor progression by modifying immune cell behavior, which creates an immunosuppressive environment to enable tumor growth and metastasis. Furthermore, EVs from tumor cells, including melanoma cells, participate in immune escape by transferring bioactive molecules between cells, thereby suppressing antitumor immune responses (33).

Collectively, these mechanisms establish an immunosuppressive tumor microenvironment (TME) that favors tumor survival, metastasis, and resistance to immunotherapy.

Melanoma cells undergo significant phenotypic transformations during this process, losing their epithelial attributes and acquiring mesenchymal traits. This transition is characterized by a reduction in E-cadherin expression, an epithelial adhesion molecule—accompanied by an increase in mesenchymal markers, including vimentin and N-cadherin. These molecular transformations improve the motility and invasiveness of melanoma cells, facilitating their dissemination to distant sites. Comprehending the underlying mechanisms of EMT in melanoma is vital for developing targeted therapies to inhibit metastasis (34). Moreover, EMT in melanoma is driven by several key signaling pathways, notably Wnt/β-catenin, transforming growth factor-beta (TGF-β), and Notch. These pathways converge on transcription factors such as Snail, Slug, and Twist, which are significant in regulating EMT. Activating these signaling cascades leads to the repression of epithelial markers like E-cadherin and the induction of mesenchymal markers, thereby increasing the motility and invasiveness of melanoma cells (9, 35). Furthermore, the tumor microenvironment (TME) significantly impacts EMT in melanoma by providing factors such as hypoxia and inflammatory cytokines. Hypoxia is a common feature in rapidly growing tumors, which stabilizes hypoxia-inducible factors (HIFs) that promote EMT by inducing mesenchymal traits and increasing resistance to apoptosis.

Similarly, inflammatory cytokines within the TME further contribute to EMT by modulating signaling pathways that suppress epithelial characteristics and promote mesenchymal attributes, thereby enhancing tumor cell invasiveness and survival (36). However, in canine oncology, such findings primarily concern oral and subungual malignant melanomas, whereas benign dermal melanocytomas exhibit limited or no metastatic behavior, emphasizing the biological contrast between these tumor types (23, 24).

### 3.3 (SCCs)

Cutaneous squamous cell carcinoma (CSCC) is a malignant tumor stemming from keratinocytes in the epidermis. In dogs, CSCC is commonly correlated with chronic exposure to UV radiation, especially in regions of poorly pigmented skin. Depending on their anatomical location, melanocytic tumors in dogs have different biological activities. While non-cutaneous melanomas, such as oral, digital, and subungual melanomas, are highly malignant, aggressive, and prone to metastasis, cutaneous melanomas are usually benign, with minimal metastatic potential and a favorable prognosis (25). These discrepancies reflect underlying differences in molecular profiles, including proliferative indices, melanin production pathways, and oncogenic marker expression. Similarly, the biological behavior of squamous cell carcinoma (SCC) varies by site of origin: cutaneous SCC is frequently associated with long-term UV exposure and typically has a better prognosis, whereas mucocutaneous and oral SCCs are more invasive and have a higher potential for metastasis (37). In canine oncology, understanding these differences is essential for precise diagnosis, prognosis, and treatment planning.

This condition is prevalent in canine breeds like Beagles, Pit Bulls, Schnauzers, Basset Hounds, Collies, and Dalmatians, who are more vulnerable to developing CSCC due to their depigmented skin (9). Primarily, UV radiation induces DNA damage by forming cyclobutane pyrimidine dimers (CPDs), which are covalent linkages between adjacent pyrimidine bases. These lesions can result in mutations in critical genes, such as the tumor suppressor gene TP53, impairing cell cycle regulation and developing carcinogenesis. The accumulation of such mutations is a primary driver of skin cancer development like SCCs. The SCC progression involves multiple molecular events, including the stimulation of oncogenes and the inactivation of tumor suppressor genes. These carcinomas frequently exhibit increased cyclooxygenase-2 (COX-2) expression, contributing to tumor growth and inflammation (38). Histologically, SCCs are characterized by invasive nests of atypical squamous cells with variable degrees of keratinization, correlating with tumor grade and prognosis. Moreover, the role of EMT has also been demonstrated in SCCs, where the loss of E-cadherin expression expedites metastasis to regional lymph nodes. In addition, syndecan-1, a transmembrane heparan sulfate proteoglycan, has been specified as a potential marker for tumor progression, with reduced expression correlating with increased invasiveness as reported for a case series for CSCC (9, 39).

It is important to emphasize that canine cutaneous SCC should be distinguished from oral or nasal SCCs, which often present distinct biological behaviors and prognostic outcomes. The cutaneous form typically develops in sun-exposed, sparsely haired, and poorly pigmented areas such as the ventral abdomen, pinnae, and nasal planum, whereas oral SCCs generally display more aggressive and infiltrative behavior. Therefore, the following discussion primarily refers to the pathophysiological features of cutaneous SCC in dogs.

SCC in dogs is a malignancy originating from keratinocytes, with its pathogenesis involving a series of molecular events that enable and drive tumor initiation and progression. Chronic UV radiation exposure is a primary etiological factor, especially in lightly pigmented dogs or those with sparse hair coats. UV-induced DNA deterioration leads to mutations in critical genes, notably the TP53 tumor suppressor gene, impairing cell cycle regulation and promoting carcinogenesis (40).

Besides TP53 mutations, the SCC progression involves molecular alterations at elementary levels. Genomic instability, characterized by elevated rates of mutations and chromosomal aberrations, has been observed in canine SCCs (41). Consequently, this instability contributes to tumor heterogeneity and evolution, facilitating the acquisition of aggressive characteristics (42).

**Figure 2:**
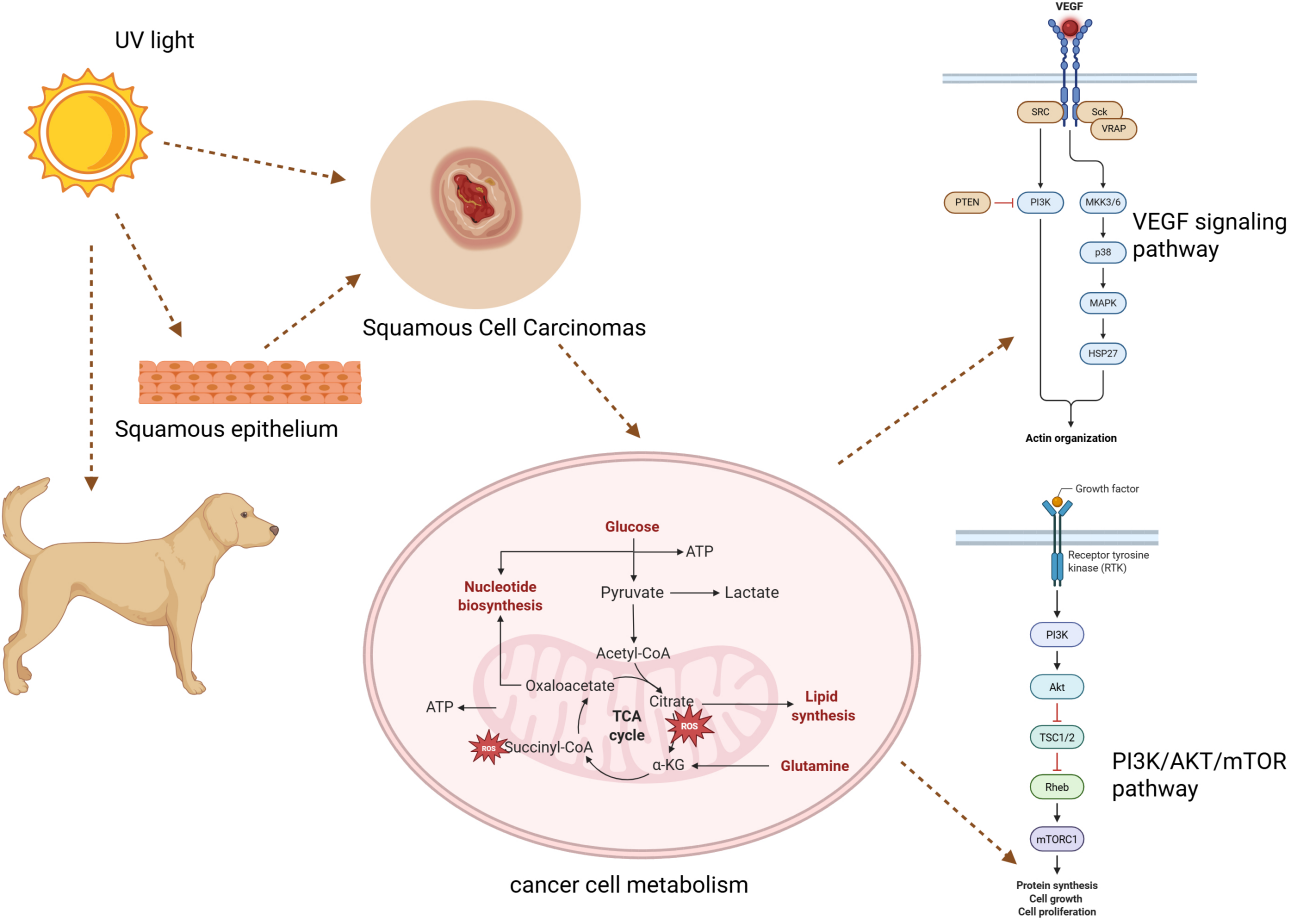
An overview of the metabolic and molecular processes behind canine squamous cell cancer. In squamous epithelium, UV light damages DNA, promoting malignant transformation. In addition to increased ROS generation and altered metabolism, cancer cells also activate the VEGF and PI3K/AKT/mTOR pathways, which promote angiogenesis, cell division, and tumor growth.

The inactivation of tumor suppressor genes like CDKN2A plays a significant role in SCC growth. For instance, the CDKN2A gene encodes the p16^INK4a^ protein, which regulates the cell cycle by inhibiting cyclin-dependent kinases. Therefore, a loss of p16^INK4a^ function leads to uncontrolled cellular proliferation, a hallmark of cancer progression. Furthermore, overexpression of the epidermal growth factor receptor (EGFR) has been reported in canine oral SCCs (38, 43). This activation triggers downstream signaling pathways, such as the PI3K/AKT pathway, promoting cell proliferation, survival, and invasion. This overexpression is correlated with increased tumor aggressiveness and poor prognosis (44) (**Figure 2**).

Although many of these molecular mechanisms have been well characterized in human cutaneous SCC, veterinary evidence indicates that similar processes underlie the pathophysiology of canine cutaneous SCC, with UV-induced TP53 mutations, COX-2 upregulation, and EGFR signaling activation as consistent findings. Moreover, vascular endothelial growth factor (VEGF) stimulates angiogenesis, improves food and oxygen delivery, and supports the metabolic needs of rapidly proliferating cancer cells in canine squamous cell carcinoma (SCC). Increased tumor cell survival, cytoskeletal remodeling, and endothelial cell proliferation result from VEGF receptor activation, which starts downstream signaling via the MAPK and PI3K/AKT/mTOR pathways. Additionally, by changing the tumor microenvironment and causing tumor-associated inflammation, VEGF promotes invasion and metastasis. In canine SCC, increased VEGF expression is associated with higher tumor grade and aggressiveness, suggesting its potential as a prognostic biomarker and therapeutic target (45). Nevertheless, canine SCC often exhibits slower metastatic behavior compared to its human counterpart, suggesting potential species-specific regulatory differences in tumor progression.

Beyond the direct genotoxic effects of UV light, the local inflammatory and oxidative environment also plays a pivotal role in promoting malignant transformation. Chronic UV exposure induces reactive oxygen species (ROS) formation and persistent inflammation, both of which can lead to DNA strand breaks, protein oxidation, and activation of pro-oncogenic signaling cascades, including NF-κB and MAPK pathways. These mechanisms collectively sustain a protumorigenic microenvironment that favors SCC development and progression.

In addition, according to recent research, canine papillomavirus (CPV) infection may act as a cofactor in the development of cutaneous squamous cell carcinoma (cSCC). CPV infection may contribute to neoplastic transformation by disrupting normal cell cycle regulation and promoting genomic instability, even though chronic ultraviolet (UV) exposure remains the predominant etiological factor. It has been demonstrated that viral oncoproteins, such as E6 and E7, inactivate tumor suppressor pathways (including p53 and pRb), promoting unchecked keratinocyte proliferation and the development of cancer (46). A growing body of research suggests that papillomavirus-associated SCCs typically occur in regions with low UV exposure and may represent a unique biological subgroup of this neoplasm, even though the precise causal association between CPV and cSCC in dogs remains unclear (47).

Similar to other epithelial malignancies, immune evasion also plays a role in canine SCC progression. Upregulation of immune checkpoint molecules, such as PD-L1, has been detected in several cutaneous epithelial tumors, potentially dampening T-cell-mediated antitumor responses. While most evidence derives from human SCC research, emerging veterinary studies suggest comparable immune escape pathways in canine SCC, warranting further investigation.

### 3.4 Emerging molecular insights

Recent advances in molecular oncology have discovered numerous biomarkers and therapeutic targets across canine skin cancers. For example, the mitotic index has emerged as a robust prognostic indicator, especially in MCTs and SCCs. Moreover, gene expression profiling has also signified novel targets, such as heat shock proteins (HSPs) and growth factor receptors, which are being explored for their therapeutic potential (48, 49).

Comparative oncology studies have revealed significant parallels between canine and human melanomas, signifying the translational value of canine models in comprehending tumor biology and developing practical, targeted therapies (50). Canine malignant melanoma (CMM), particularly in the oral cavity, shares striking similarities with human mucosal melanoma, including aggressive behavior, metastatic patterns, and resistance to traditional treatment plans. These shared attributes make CMM a valuable spontaneous tumor model for studying human melanoma subtypes (51).

Advances in molecular oncology have specified multiple biomarkers and therapeutic targets in canine skin cancers. For instance, the mitotic index is a well-established prognostic indicator, particularly in MCTs. Studies have reported that dogs with MCTs presenting a mitotic index greater than 5 have a median survival time of 3 months, compared to 80 months for those with a mitotic index of 5 or less (2).

Gene expression profiling has revealed novel targets, including heat shock proteins (HSPs) and growth factor receptors. Overexpression of HSPs, including HSP27, HSP72, and HSP73, has been observed in different canine skin tumors, implying their role in tumor progression and potential as therapeutic targets (48). Similarly, growth factor receptors like EGFR have been implicated in tumor proliferation and resistance mechanisms, making them promising candidates for targeted therapies (52).

As discussed above, comparative oncology studies have underlined significant parallels between canine and human melanomas, particularly associated with shared molecular modifications. For instance, mutations in the BRAF, NRAS, and KIT genes are well-documented in human melanomas, leading to constitutive activation of the MAPK pathways promoting tumorigenesis. Likewise, canine melanomas exhibit mutations in these genes with varying frequencies. For example, BRAF mutations, prevalent in approximately 50% of human melanomas, are less frequent in canine melanomas, occurring in ~6% of cases (6). Moreover, NRAS mutations have been identified in both human and canine melanomas, contributing to tumor development in both species. KIT mutations, related to specific subtypes of human melanomas, have also been reported in canine melanomas. These shared molecular features signify the translational value of canine models for comprehending melanoma biology and cultivating novel therapeutic strategies (22, 53).

Beyond canonical oncogenic mutations, recent transcriptomic and proteomic analyses have revealed that immune-related gene alterations—such as upregulation of PD-L1, downregulation of MHC class I molecules, and altered cytokine signaling networks—play central roles in defining the aggressiveness of canine melanomas. Integrating molecular and immunologic profiling will be crucial for developing predictive biomarkers and individualized immunotherapy strategies in veterinary oncology (54).

### 3.5 Diagnostic approaches in canine skin cancers

Skin Accurate diagnosis of canine skin cancers is vital for effective treatment plans and prognosis. Traditional methods such as cytology and histopathology remain the gold standard, while advanced molecular and imaging techniques continuously improve diagnostic precision and therapeutic decision-making.

Fine-needle aspiration cytology (FNAC) is a minimally invasive diagnostic methodology broadly utilized in veterinary medicine to evaluate cell morphology and offer quick preliminary diagnoses. This method is immensely beneficial in distinguishing between inflammatory, benign, and malignant lesions. FNAC is typically utilized to evaluate cutaneous and subcutaneous masses, enlarged lymph nodes, and other accessible structures (55). The FNAC procedure involves using a fine-gauge needle, with or without suction, to collect cellular material from the lesion. Subsequently, the collected sample is spread onto a glass slide, stained, and examined under a microscope to evaluate cellular characteristics. The procedure offers multiple benefits, such as cost-effectiveness and easy execution. More significantly, it typically does not require sedation or anesthesia. However, it is imperative to realize FNAC’s limitations, such as the potential for non-diagnostic samples or the inability to evaluate tissue architecture fully. In some cases, supplementary diagnostic procedures like histopathology may be essential for a definitive diagnosis (56). However, FNAC shows limitations in tumor grading and cannot reliably assess parameters such as tissue architecture, depth of invasion, and mitotic index. Therefore, histopathological assessment becomes imperative for definitive diagnosis (55, 56).

Histopathological assessment is the gold standard for diagnosing and grading tumors, as it allows for evaluating critical parameters like mitotic index, invasion depth, and tissue architecture. These evaluations are vital for determining tumor behavior and guiding treatment methods. Moreover, immunohistochemical (IHC) staining further improves diagnostic accuracy by detecting specific molecular markers related to various tumor types. For instance, c-KIT (CD117) expression is vital for classifying MCTs, while markers like Melan-A and S100 are essential for diagnosing melanomas (57). These molecular markers help differentiate melanomas from other malignancies and benign lesions. By integrating histopathological assessment with IHC staining, pathologists can accomplish a more precise diagnosis and prognosis and design advanced targeted therapeutic strategies.

Recent innovations in molecular diagnostics have further refined cancer classification methods. For example, next-generation sequencing (NGS), polymerase chain reaction (PCR)-based mutation analysis, and fluorescence *in situ* hybridization (FISH) can identify genetic mutations and chromosomal aberrations correlated with aggressive tumor behavior (22). Furthermore, advancements in molecular oncology have facilitated the detection of mutations in genes like BRAF, NRAS, and c-KIT in canine melanomas. These advanced methods offer in-depth insights for developing targeted therapies. For instance, Mochizuki et al. (2015) found a BRAF mutation in 6% of canine melanomas, signifying the potential for targeted therapies in such cases (22).

Advanced imaging modalities, including CT scans, magnetic resonance imaging (MRI), and positron emission tomography (PET, have become critical tools for tumor staging and metastasis detection. In particular, CT scans are valuable in evaluating deep-seated tumors and bone invasion, while MRI offers superior soft tissue contrast, aiding in surgical planning (58). Moreover, PET imaging, mainly when combined with computed tomography (PET/CT) or magnetic resonance imaging (PET/MRI), is valuable in evaluating tumor metabolism and detecting early metastatic spread in aggressive malignancies like melanomas and soft tissue sarcomas. By employing radiotracers like fluorine-18 fluorodeoxyglucose (F-18 FDG), PET imaging helps evaluate the metabolic activity of tumors, offering valuable insights into tumor aggressiveness and assisting in staging and treatment planning (59).

## 4 Therapeutic approaches for canine skin cancer

### 4.1 Overview of treatment modalities

Treatment options for canine skin cancer depend on the tumor’s type, grade, stage, and the overall health of the dog. The primary goal of therapy is to cure the disease or provide palliation to prolong survival and improve quality of life (QoL). Typical treatment modalities include surgical removal, radiation therapy, chemotherapy, and immunotherapy (59). The treatment choice is customized to each case, incorporating factors such as tumor location, size, and metastatic potential. Early detection and appropriate therapeutic interventions are vital for achieving favorable outcomes in dogs with skin cancer. In many cases, multimodal approaches integrating chemotherapy, radiation therapy, and immunotherapy are utilized to optimize outcomes (60). Nonetheless, key factors influencing therapeutic decisions include the tumor’s biological behavior, its metastatic potential, and the dog’s age and comorbidities. Early-stage tumors are often more amenable to aggressive treatment plans, whereas advanced-stage malignancies may require palliative methods to manage symptoms and prolong survival (61).

### 4.2 Radiation therapy

Radiation therapy is a vital element of managing canine skin cancer, particularly for non-resectable or incompletely excised tumors. It is typically employed for MCTs, melanomas, and SCCs, with high efficacy in controlling local tumor growth (62, 63). Traditional fractionation protocols involve delivering small doses of radiation over multiple sessions, minimizing the risk of toxicity to surrounding tissues (64).

However, it should be noted that radiation therapy is infrequently used for cutaneous squamous cell carcinoma (SCC) and cutaneous malignant melanomas in dogs. Its application is generally reserved for more invasive or non-resectable variants, such as subungual melanomas and digit-associated SCCs, where surgical margins are challenging to achieve or recurrence is likely (24).

A newer modality, Stereotactic radiation therapy (SRT), delivers high doses of radiation in fewer sessions with greater precision. This approach has demonstrated excellent promise in treating aggressive melanomas and high-grade MCTs, reducing tumor spread significantly while minimizing adverse effects. In the context of canine cutaneous cancers, SRT has primarily been explored for deeply invasive or subungual melanomas rather than benign cutaneous melanocytomas or superficial SCCs, which are typically managed surgically (24). However, potential side effects, such as dermatitis, alopecia, and fibrosis, must be closely monitored (65).

### 4.3 Dose protocols and examples

Definitive radiation therapy is administered with curative intent, typically involving daily treatments from Monday through Friday for 3–4 weeks, amounting to 15–20 sessions. Each fraction comprises a small calculated dose to optimize tumor control while minimizing adverse impacts on surrounding tissues. A canine patient with an incompletely excised MCT may undergo 15 daily radiation therapy sessions. Research studies have demonstrated tumor control rates exceeding 90% in such cases when definitive protocols are implemented (Veterinary Specialty Center, 2022).

For cutaneous SCC and cutaneous malignant melanomas, definitive radiation therapy is seldom performed due to the generally limited radiosensitivity of these tumor types. When used, it is typically applied to incompletely excised or non-resectable subungual and oral melanomas or digit-associated SCCs rather than to dermal cutaneous lesions (2).

Palliative radiation therapy is designed to relieve pain and discomfort in cases of advanced malignancy or when comorbidities preclude multiple anesthesia events. This approach involves a lower total radiation dose with more significant fractions, administered daily over one week or once weekly for four weeks, depending on the patient’s condition and therapeutic objectives (Iowa Veterinary Specialties, n.d.). In a canine patient with a non-resectable tumor causing substantial discomfort, a palliative protocol may include a weekly radiation therapy session for four consecutive weeks, prioritizing symptom relief rather than tumor eradication (VCA Animal Hospitals, n.d.).

Stereotactic radiation therapy (SRT) is an advanced modality that delivers high radiation doses in fewer sessions with better precision, enabling targeted treatment of neoplastic tissues (65). Moreover, these protocols typically involve 1–5 treatment sessions, each delivering a higher radiation dose per fraction than traditional fractionation schedules. SRT has been utilized in treating aggressive melanomas and high-grade MCTs, substantially containing tumors and minimizing adverse effects. In canine oncology, its use has been most beneficial for subungual and oral malignant melanomas or locally invasive SCCs where surgery alone cannot achieve local control. There is currently limited evidence supporting the routine use of SRT for benign or superficial cutaneous tumors (24). Potential side effects such as dermatitis, alopecia, and fibrosis require close monitoring to ensure optimal patient outcomes (65).

### 4.4 Chemotherapy

Chemotherapy is critical in managing systemic or high-grade tumors in dogs. For MCTs, vinblastine and lomustine are typically utilized chemotherapeutic agents. These drugs target rapidly dividing cells, reducing tumor burden in aggressive cases. Research has demonstrated the efficacy of vinblastine in treating canine MCTs, with significant tumor reduction observed in treated dogs. Likewise, lomustine has exhibited effective management of high-grade MCTs, often in combination with prednisone (66).

Corticosteroids like prednisone are frequently merged with chemotherapy to mitigate inflammation and reduce tumor-associated edema. Prednisone has been demonstrated to minimize swelling around tumors, making surgical removal more effective. In one study, prednisone reduced tumor size by 25% in over 80% of dogs with MCTs, although this effect was temporary (66).

Chemotherapy is also employed for cutaneous variants of canine melanoma and SCC, although its use is generally limited due to the lower aggressiveness of dermal cutaneous forms compared to oral or subungual tumors. In cutaneous melanoma, therapy differs between benign dermal hair-skinned melanomas and more malignant subungual (digit-associated) melanomas, the latter often requiring more aggressive protocols (23, 24). For cutaneous SCCs, chemotherapy is typically reserved for non-resectable or locally invasive tumors rather than superficial lesions.

Additionally, electrochemotherapy has been reported as an alternative therapy for canine cutaneous SCC, particularly in cases of non-resectable or locally advanced tumors, and should be considered alongside conventional chemotherapy (67).

Chemotherapy is a cornerstone in managing various malignancies, including melanoma and SCCs. In cases of metastatic or unresectable melanoma, *dacarbazine* has remained an enduring chemotherapy drug used in treatment. It is an alkylating agent that results in DNA adducts, leading to cytotoxic impacts on malignant cells. For SCCs, mainly when surgical or radiotherapeutic interventions are not viable, palliative chemotherapy with cisplatin or carboplatin is effective. A study comparing chemoradiation with cisplatin versus carboplatin for SCCs of the head and neck determined no significant differences in loco-regional control, metastases-free survival, overall survival, and toxicities between the two agents. These results suggest that carboplatin is a reasonable alternative for patients who cannot receive cisplatin. While chemotherapy can extend survival, its usage is often limited due to its side effects, such as myelosuppression, gastrointestinal disturbances, and hepatotoxicity. Supportive therapies, including anti-nausea medications and regular bloodwork monitoring, are essential to improve tolerability (68).

Chemotherapy is critical in managing systemic or high-grade tumors in dogs. For MCTs, vinblastine is administered intravenously at a dosage of 2 mg/m², usually every week. Similarly, lomustine is given orally at 70 to 90 mg/m² every 3 to 6 weeks, depending on the specific protocol and patient tolerance (69). These agents target rapidly dividing cells, reducing tumor burden in aggressive cases. Corticosteroids, such as prednisone, are often integrated into chemotherapy to mitigate inflammation and minimize tumor-associated edema. Prednisone is typically administered at anti-inflammatory doses of 0.5 to 1 mg/kg/day (70).

For cutaneous melanoma, chemotherapy such as carboplatin may be used primarily in subungual or non-resectable variants rather than benign dermal forms. Carboplatin is administered intravenously at 300 mg/m² every 3 weeks, with adjustments based on renal function and patient size (23). For cutaneous SCCs, palliative chemotherapy with cisplatin or carboplatin is considered for locally invasive or non-resectable lesions, typically at doses of 50–70 mg/m² every 3 weeks depending on patient size and condition (70).

Electrochemotherapy is another reported treatment modality for canine cutaneous SCC and may be considered in select cases where conventional chemotherapy or surgery is not feasible (67). While chemotherapy can prolong survival, side effects like myelosuppression, gastrointestinal disturbances, and hepatotoxicity often limit its usage. Supportive therapies can improve tolerance, including anti-nausea medications and regular bloodwork monitoring.

Overall, the use of chemotherapy in canine cutaneous melanoma and SCC is far more limited compared to oral or digit-associated tumors, and protocols should be tailored to the tumor location, invasiveness, and histologic grade.

### 4.5 Immunotherapy

Immunotherapy represents one of the most dynamic and rapidly advancing treatment strategies in veterinary oncology, especially for canine malignant melanoma. Its core principle lies in harnessing and amplifying the host immune system to recognize and destroy tumor cells while minimizing systemic toxicity. In recent years, immunotherapy has emerged as an innovative approach to managing canine skin cancer, particularly for melanomas (71). The canine melanoma vaccine (Oncept), which utilizes human tyrosinase as an immunogenic target, has shown variable efficacy in clinical studies. Recent reviews indicate that vaccine-induced immune responses are limited, with T cell responses primarily observed in healthy Beagles and only modest antibody responses detected in canine melanoma patients (72). Therefore, contrary to some earlier reports, a “robust immune response” in vaccinated melanoma patients has not been demonstrated. The Oncept vaccine is typically investigated for oral malignant melanomas, which are more aggressive than cutaneous variants, and its use in cutaneous melanoma remains largely investigational (72).

DNA vaccines targeting chondroitin sulfate proteoglycan 4 (CSPG4) are another immunotherapeutic approach under investigation for canine melanoma. These vaccines aim to stimulate immune responses against CSPG4-expressing melanoma cells and are being evaluated for both cutaneous and oral malignant melanomas (72). Early studies suggest CSPG4 vaccines may induce measurable antibody and T-cell responses in vaccinated dogs, representing a promising avenue for further research.

Checkpoint inhibitors, particularly anti-PD-L1 antibodies, are under investigation for canine melanoma. These agents block immune checkpoint pathways, thereby restoring T-cell-mediated antitumor immunity (72). Preliminary studies have produced promising results. For instance, Maekawa et al. (2023) evaluated the safety and effectiveness of a canine chimeric anti-PD-L1 antibody (c4G12) in dogs with advanced malignant tumors, including melanoma. In some cases, the study reported partial responses, indicating potential clinical benefits (72, 73).

Emerging therapies, *e.g.*, cytokine-based immunotherapy and dendritic cell vaccines, offer promising avenues for improving antitumor immune responses in canine cancers. Among them, cytokine-based immunotherapy involves administering immunostimulatory cytokines, such as interleukin-2 (IL-2) and interferon-alpha (IFN-α), which are integral to modulating the immune system’s response to tumors. Moreover, IL-2 promotes the proliferation and activation of cytotoxic T lymphocytes and natural killer cells, while IFN-α improves antigen presentation and the cytotoxic activity of immune cells. Several studies have investigated these cytokines for their anticancer properties, with some researchers indicating their potential to induce tumor regression and enhance survival rates in various cancers (74). The ability of cytokine-based tactics, such as GM-CSF-expressing vectors and IL-15 superagonists, to promote dendritic cell activation and cytotoxic lymphocyte infiltration is being investigated. Moreover, advances in adoptive cell transfer — such as autologous lymphokine-activated killer (LAK) cells and engineered T-cell therapies — represent emerging directions in canine immunotherapy research (75).

### 4.6 Targeted therapies

Targeted therapies have transformed the treatment of specific canine skin cancers by focusing on molecular pathways critical to tumor survival and progression. In MCTs, tyrosine kinase inhibitors (TKIs) like toceranib phosphate (Palladia) and masitinib selectively inhibit c-KIT signaling, thereby controlling tumors with KIT mutations. Mutations in the c-kit proto-oncogene correlate with the tumorigenesis of MCTs, resulting in growth factor-independent and constitutive phosphorylation of the KIT receptor tyrosine kinase. Toceranib phosphate has exhibited biological activity against MCTs, with studies indicating that tumors harboring c-kit mutations exhibit an increased objective response rate to TKI therapy (76). Clinical trials have significantly improved progression-free survival for dogs treated with TKIs. For instance, a randomized, placebo-controlled phase III clinical trial evaluating masitinib in dogs with non-metastatic, recurrent, or non-resectable grade II or III MCTs reported a median time to tumor progression of 118 days in the masitinib group, compared to 75 days in the placebo group.

Similarly, a multi-center, placebo-controlled, double-blind, randomized study determined the effectiveness of toceranib phosphate (Palladia) in treating dogs with recurrent MCTs. The study found that dogs receiving toceranib had a significantly higher objective response rate than the corresponding placebo group, indicating a delay in disease progression (76).

Targeted therapies against BRAF and NRAS mutations have been recently explored in melanomas. These mutations activate the MAPK signaling pathway, promoting uncontrolled cell proliferation. Drugs targeting these pathways potentially reduce tumor size and delay metastasis. Likewise, COX-2 inhibitors, such as piroxicam, have demonstrated efficacy in SCCs by suppressing inflammation and tumor progression (77). Personalized medicine, driven by genetic and molecular profiling processes, is expected to further refine targeted therapy approaches, customizing treatments to individual tumors and enhancing outcomes (78).

### 4.7 Palliative care and quality of life

Effective pain control often incorporates a multimodal approach, integrating various pharmacologic agents to address multiple pain pathways. Among various pain control methods, nonsteroidal anti-inflammatory drugs (NSAIDs) are frequently used to reduce inflammation and relieve pain. In the case of more severe pain, opioids may be administered. In addition, adjunctive therapies such as gabapentin or amantadine can be incorporated to manage neuropathic pain components. This thorough strategy offers optimal analgesia for canine cancer patients. Maintaining proper nutrition is vital for dogs battling cancer. Moreover, diets rich in protein and fat can help preserve lean body mass and offer necessary energy (79). Supplementing with omega-3 fatty acids, such as eicosapentaenoic acid (EPA) and docosahexaenoic acid (DHA), can reduce inflammation and may slow tumor progression. In addition, antioxidants can support overall health by neutralizing free radicals and minimizing oxidative stress. In the terminal cancer stages, hospice care prioritizes comfort and dignity. Customized interventions may include fluid therapy to prevent dehydration, wound management to tackle ulcerated tumors, and counseling for pet owners to support decision-making and emotional well-being. This inclusive approach ensures that a dog’s remaining time is as comfortable and fulfilling as possible (80).

### 4.8 Emerging therapies and future directions

Veterinary oncology has rapidly evolved, with emerging therapies offering new hope for canine skin cancer patients. For instance, nanomedicine, involving nanoparticle-based drug delivery systems, improves the precision and efficacy of chemotherapy and targeted therapies while minimizing systemic toxicity (81). Gene therapy, particularly CRISPR-based approaches, can potentially correct genetic mutations related to tumorigenesis. Early studies in canine models have demonstrated the feasibility of gene-editing methods in targeting cancer-causing pathways (82). Furthermore, comparative oncology, leveraging the similarities between canine and human cancers, continues to drive novel diagnostic and treatment methodologies in diagnostics and therapeutics. Collaborative clinical trials involving both species are accelerating the development of novel therapies with promising implications for both veterinary and human medicine (83).

Future directions in canine skin cancer therapy increasingly focus on the convergence of nanotechnology and immunotherapy. Nanoparticle-based delivery systems are being developed to transport cytokines, peptides, or checkpoint inhibitors directly into the tumor microenvironment, enhancing therapeutic efficacy while minimizing off-target effects (54).

### 4.9 Challenges and limitations

Despite advancements, several challenges exist in treating canine skin cancer. For example, financial constraints often limit access to advanced therapies, especially in resource-limited settings (84). In addition, ethical considerations originate when using experimental treatments, accentuating the need for informed consent and transparency in clinical trials (84). Long-term monitoring for treatment-related side effects, such as cardiotoxicity or renal dysfunction, is imperative to ensure safety and efficacy. Further research is required to develop cost-effective and accessible treatment options that meet the diverse needs of veterinary patients (85).

Research on immunotherapy in veterinary oncology has grown quickly, yet there are still obstacles to overcome. These include the high cost of producing antibodies for specialized veterinary markets, regulatory obstacles that postpone product approval, and the restricted availability of biologic medicines unique to dogs. Furthermore, variability in tumor antigen expression and immune responses across breeds complicates the establishment of universal therapeutic protocols (86).

## 5 Comparative oncology: bridging human and veterinary medicine

### 5.1 Role of dogs in translational research

Dogs are increasingly used as models for preclinical and translational studies, particularly for testing novel techniques like immunotherapy and targeted treatments (87). Clinical trials in dogs often provide early insights into efficacy and safety profiles, accelerating treatment designs for both veterinary and human applications (88). Moreover, their shorter lifespans allow for faster observation of long-term treatment outcomes (89). Multiple large-scale comparative oncology studies have shown the viability of canine models in evaluating checkpoint inhibitors and cancer vaccines. For instance, canine osteosarcoma has been broadly explored due to its similarities with pediatric osteosarcoma in humans to identify new immunotherapeutic targets (90). Moreover, spontaneous canine brain tumors are now being utilized to design and refine glioblastoma treatments for humans, as they mimic the molecular heterogeneity witnessed in human patients (90).

### 5.2 Case studies and breakthroughs

Multiple innovative therapies have emerged from comparative oncology research. For instance, developing TKIs for MCTs in dogs paved the way for similar treatments in human oncology. Furthermore, studies on the canine melanoma vaccine have informed strategies for developing human cancer vaccines (90). A remarkable case study involves using oncolytic virotherapy in canine patients. Researchers investigating the efficacy of modified viruses in treating aggressive tumors found that canine trials offered vital data on dosing, immune response, and enduring safety, which were later translated into human clinical trials (91). Moreover, research on canine hemangiosarcoma, a highly aggressive cancer, has led to novel insights into angiogenesis inhibitors that could benefit human patients with vascular tumors (91).

### 5.3 Ethical and practical considerations

While comparative oncology holds immense promise, it raises ethical issues regarding the use of companion animals in research. Therefore, it is imperative to ensure informed consent, minimize discomfort, and prioritize animal welfare while conducting this research (92). In addition to ethical considerations, practical challenges exist in performing large-scale comparative oncology trials. Several factors, including breed-specific cancer predisposition, genetic diversity, and owner compliance, can affect study outcomes and data interpretation (14). Therefore, addressing these challenges requires standardized protocols and greater collaboration between veterinary and human oncologists to ensure valuable and reproducible results.

## 6 Future perspectives

Even though we now know much more about canine skin malignancies, such as mast cell tumors, melanomas, and squamous cell carcinomas, a few new molecular factors remain to be investigated. Recent research has highlighted the significance of extrachromosomal DNA (ecDNA) in tumor evolution and treatment resistance. These circular DNA segments, which include regulatory sequences or oncogenes, provide dynamic genomic plasticity and intratumoral heterogeneity (93). Therefore, examining ecDNA profiles in canine cancers may yield new prognostic and treatment-response indicators.

Similarly, changes in mitochondrial DNA (mtDNA) have attracted greater attention due to their links to cancer metabolism, oxidative stress control, and metastatic behavior. Novel approaches to diagnosis and treatment may be enabled by understanding mtDNA mutations and copy number changes in canine neoplasms (94).

Additionally, one intriguing but little-studied area of veterinary oncology is monoclonal antibody (mAb) therapy. Preliminary research on targeted immunotherapies targeting specific tumor-associated antigens, such as PD-L1 or KIT, has yielded promising results. Increasing research into canine-specific or cross-reactive monoclonal antibodies may significantly enhance the effectiveness and precision of skin cancer treatments (95).

By bridging the gap between basic research and clinical application, further integration of these new molecular and immunologic insights will improve our capacity to identify, classify, and treat canine skin malignancies.

## 7 Conclusion

Comparative oncology is a critical bridge between veterinary and human cancer studies, providing a unique opportunity to explore naturally occurring tumors in dogs and apply the outcomes to human medicine. This field has contributed significantly to exploring cancer genetics, tumor growth, and therapeutic response. Consequently, they advance targeted therapies, immunotherapies, and translational research models. The shared molecular and genetic attributes between canine and human cancers highlight the value of using dogs as models in oncological studies. Genetic parallels, such as BRAF and TP53 mutations, have allowed scientists to refine targeted treatments and specify potential biomarkers for early diagnosis. Moreover, the rapid development of canine clinical trials has offered an in-depth insight into the safety and efficacy of novel cancer treatment methods, accelerating their translation into human medicine. Despite the countless benefits of comparative oncology, multiple challenges remain to utilize its benefits. These challenges include ethical concerns, funding limitations, and logistical impediments in conducting large-scale veterinary clinical trials. Therefore, addressing these issues requires stronger collaboration between veterinary and human oncologists. Moreover, increased public awareness and financial support are essential for future research efforts. In the future, comparative oncology can yield promising outcomes through continued advancements in genetic profiling, precision medicine, and immunotherapeutic methods. By leveraging these data-driven technologies, researchers can improve human and animal cancer outcomes, reinforcing the importance of a *One Health* approach in cancer research.

## Glossary

BRAF: v-Raf murine sarcoma viral oncogene homolog B
CDKN2A: Cyclin-dependent kinase inhibitor 2A
COX-2: Cyclooxygenase-2
CT: Computed tomography
CTL: Cytotoxic T lymphocyte
CTLA-4: Cytotoxic T-lymphocyte-associated protein 4
DNA: Deoxyribonucleic acid
EGFR: Epidermal growth factor receptor
EMT: Epithelial-to-mesenchymal transition
FISH: Fluorescence *in situ* hybridization
FNAC: Fine-needle aspiration cytology
HIF: Hypoxia-inducible factor
HSP: Heat shock protein
IL-10: Interleukin-10
ITD: Internal tandem duplication
KIT: Stem cell factor receptor proto-oncogene
MAPK: Mitogen-activated protein kinase
MCT: Mast cell tumor
MDSC: Myeloid-derived suppressor cell
MHC: Major histocompatibility complex
MMP: Matrix metalloproteinase
MRI: Magnetic resonance imaging
NGS: Next-generation sequencing
NK: Natural killer (cell)
NRAS: Neuroblastoma RAS viral oncogene homolog
NSAID: Nonsteroidal anti-inflammatory drug
PD-1: Programmed cell death protein 1
PD-L1: Programmed death-ligand 1
PET: Positron emission tomography
RNA: Ribonucleic acid
ROS: Reactive oxygen species
SCC: Squamous cell carcinoma
SRT: Stereotactic radiation therapy
TAM: Tumor-associated macrophage
TGF-β: Transforming growth factor-beta
TIL: Tumor-infiltrating lymphocyte
TKI: Tyrosine kinase inhibitor
TME: Tumor microenvironment
Treg: Regulatory T cell
UV: Ultraviolet
VEGF: Vascular endothelial growth factor
